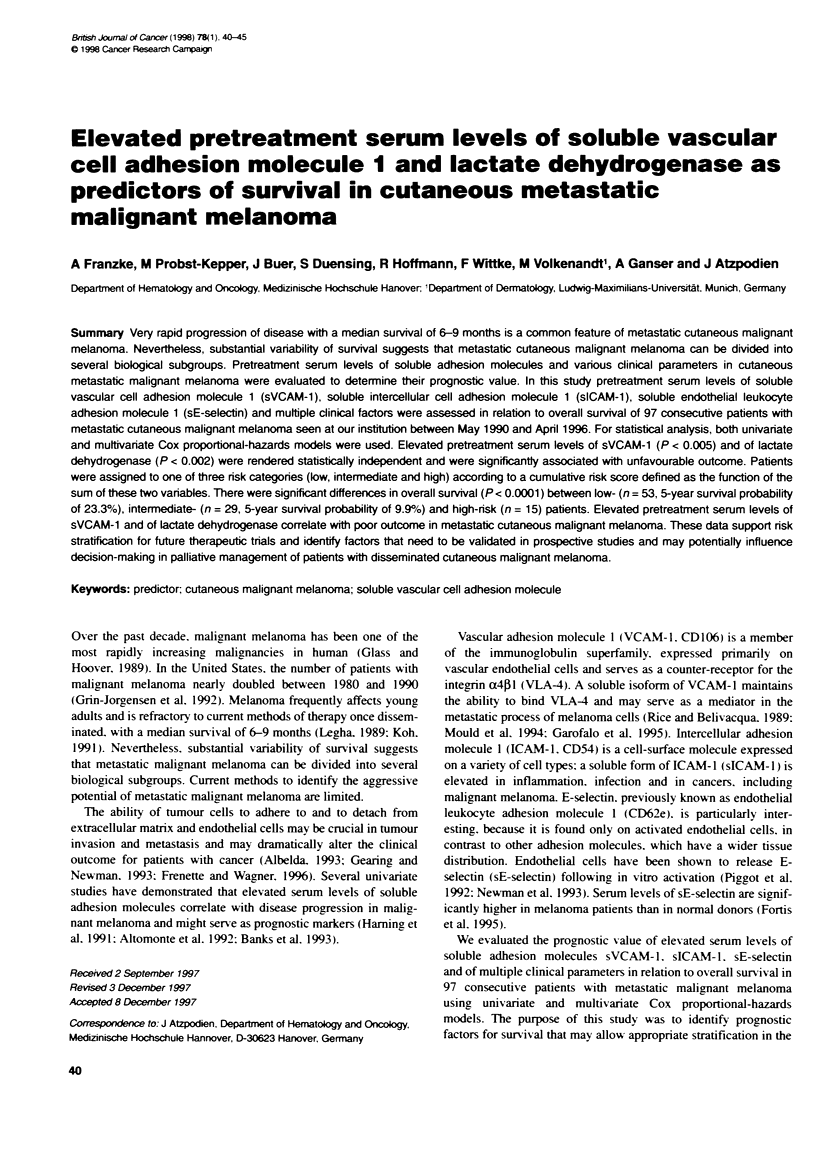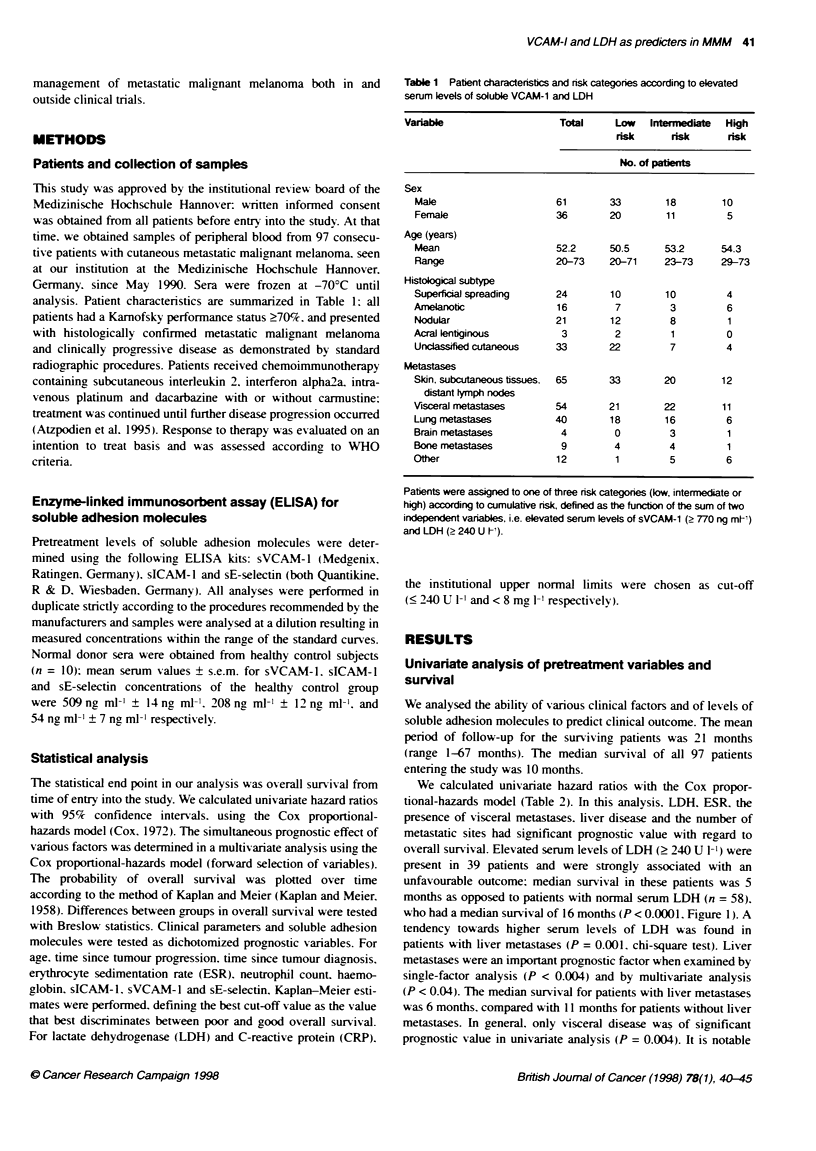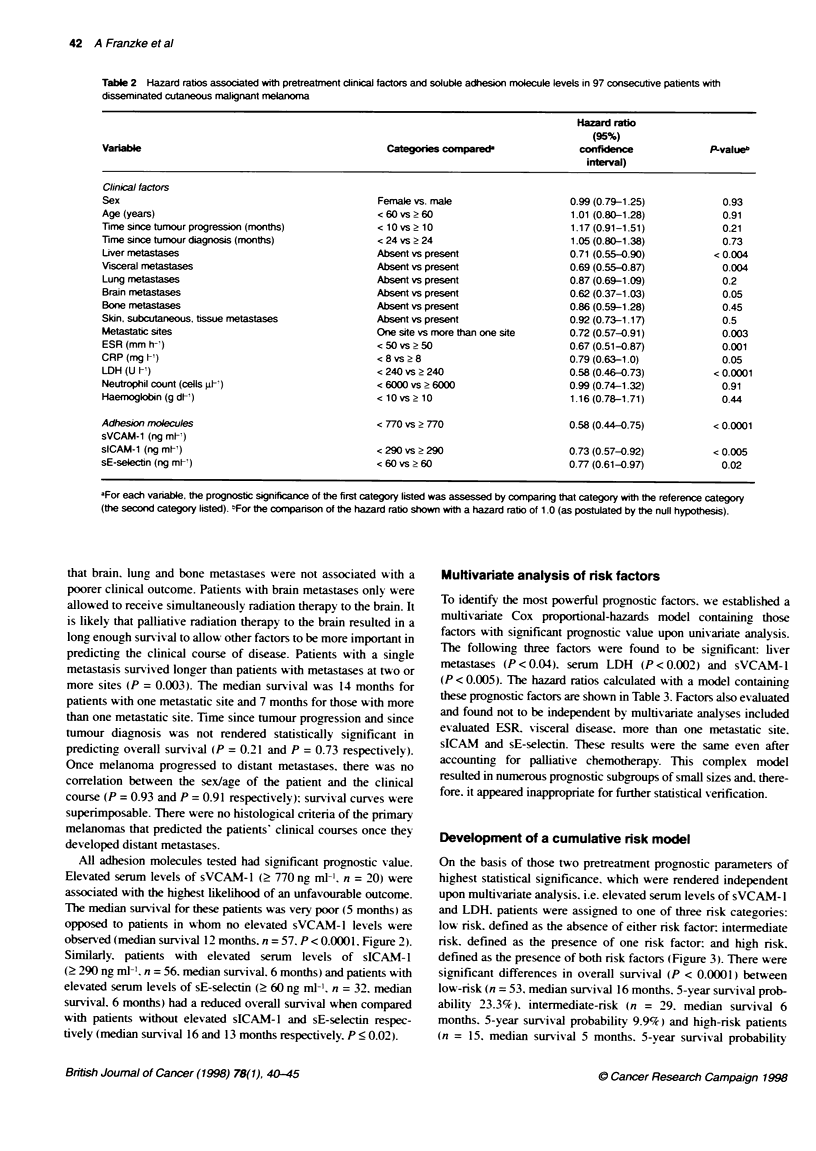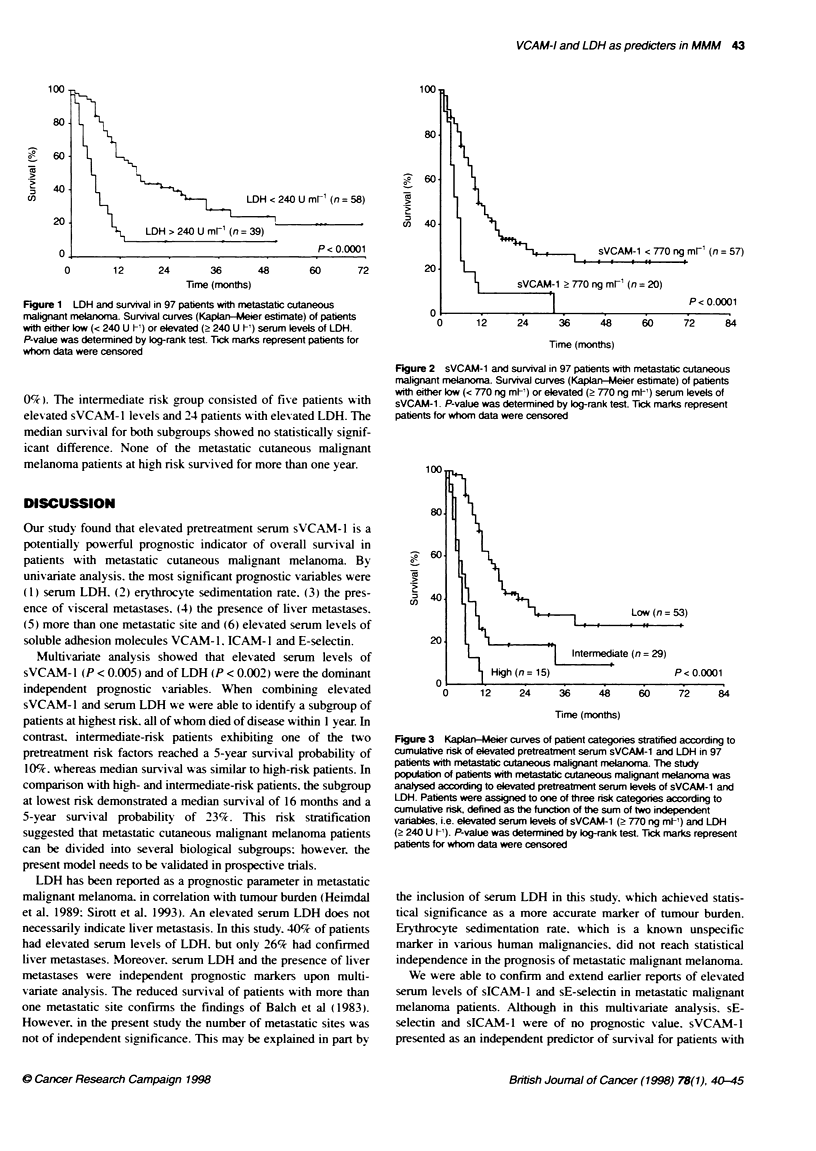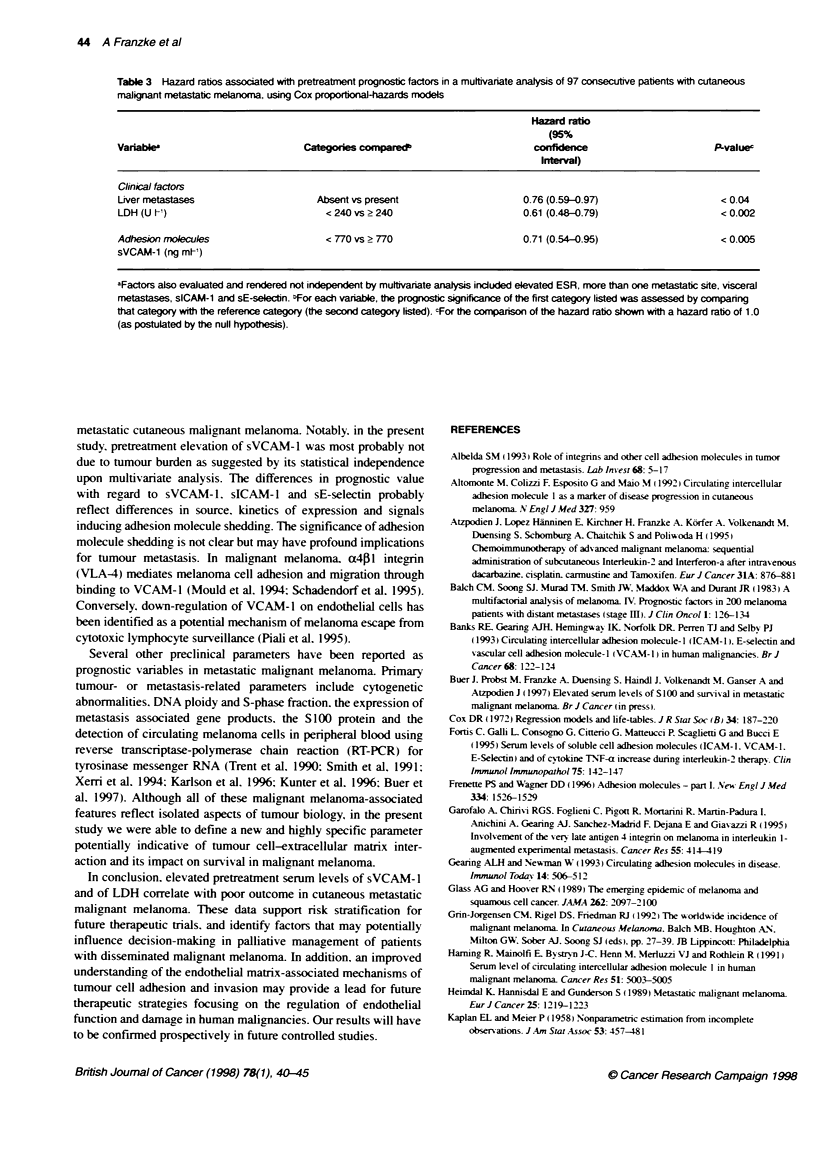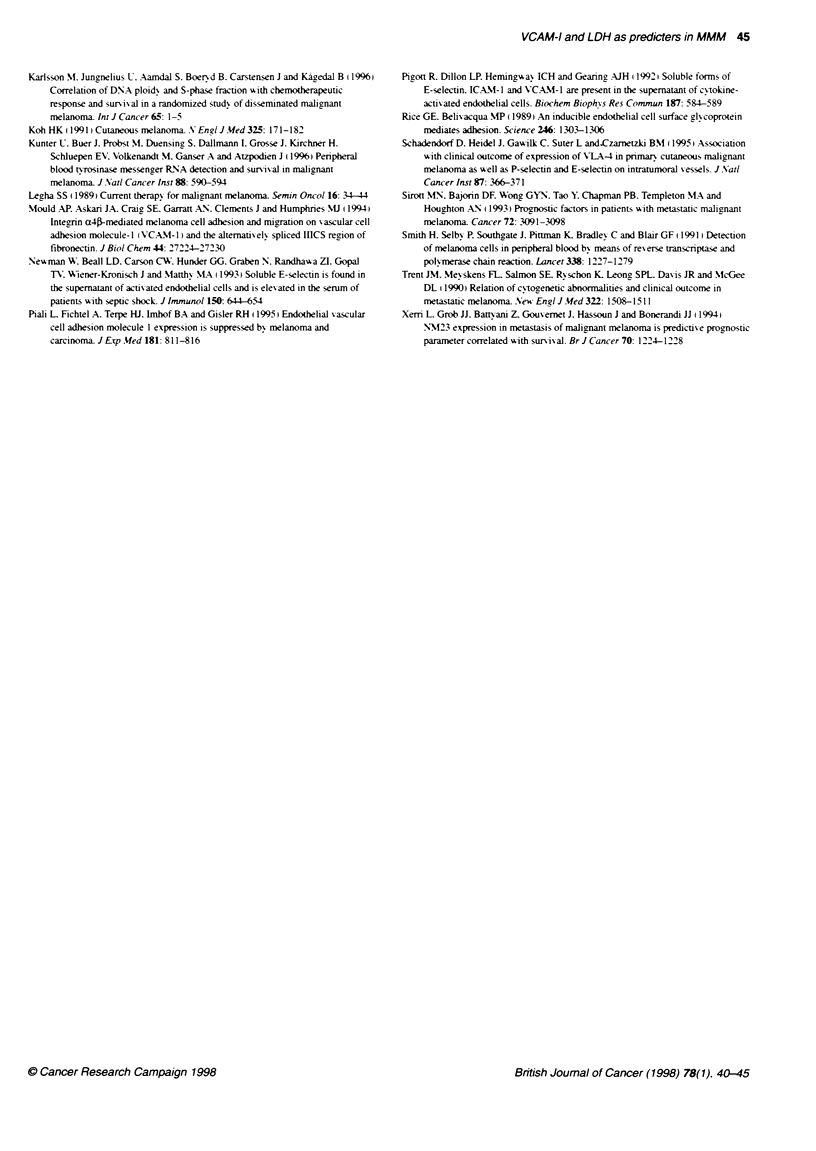# Elevated pretreatment serum levels of soluble vascular cell adhesion molecule 1 and lactate dehydrogenase as predictors of survival in cutaneous metastatic malignant melanoma.

**DOI:** 10.1038/bjc.1998.439

**Published:** 1998-07

**Authors:** A. Franzke, M. Probst-Kepper, J. Buer, S. Duensing, R. Hoffmann, F. Wittke, M. Volkenandt, A. Ganser, J. Atzpodien

**Affiliations:** Department of Hematology and Oncology, Medizinische Hochschule Hanover, Germany.

## Abstract

Very rapid progression of disease with a median survival of 6-9 months is a common feature of metastatic cutaneous malignant melanoma. Nevertheless, substantial variability of survival suggests that metastatic cutaneous malignant melanoma can be divided into several biological subgroups. Pretreatment serum levels of soluble adhesion molecules and various clinical parameters in cutaneous metastatic malignant melanoma were evaluated to determine their prognostic value. In this study pretreatment serum levels of soluble vascular cell adhesion molecule 1 (sVCAM-1), soluble intercellular cell adhesion molecule 1 (sICAM-1), soluble endothelial leukocyte adhesion molecule 1 (sE-selectin) and multiple clinical factors were assessed in relation to overall survival of 97 consecutive patients with metastatic cutaneous malignant melanoma seen at our institution between May 1990 and April 1996. For statistical analysis, both univariate and multivariate Cox proportional-hazards models were used. Elevated pretreatment serum levels of sVCAM-1 (P < 0.005) and of lactate dehydrogenase (P < 0.002) were rendered statistically independent and were significantly associated with unfavourable outcome. Patients were assigned to one of three risk categories (low, intermediate and high) according to a cumulative risk score defined as the function of the sum of these two variables. There were significant differences in overall survival (P < 0.0001) between low- (n = 53, 5-year survival probability of 23.3%), intermediate- (n = 29, 5-year survival probability of 9.9%) and high-risk (n = 15) patients. Elevated pretreatment serum levels of sVCAM-1 and of lactate dehydrogenase correlate with poor outcome in metastatic cutaneous malignant melanoma. These data support risk stratification for future therapeutic trials and identify factors that need to be validated in prospective studies and may potentially influence decision-making in palliative management of patients with disseminated cutaneous malignant melanoma.


British Journal of Cancer (1998) 78(1). 40–45
© 1998 Cancer Research Campaign

### Elevated pretreatment serum levels of soluble vascular cell adhesion molecule 1 and lactate dehydrogenase as predictors of survival in cutaneous metastatic malignant melanoma

**A Franzke, M Probst-Kepper, J Buer, S Duensing, R Hoffmann, F Wittke, M Volkenandt[1], A Ganser and J Atzpodien**

Department of Hematology and Oncology, Medizinische Hochschule Hanover; [1]Department of Dermatology, Ludwig-Maximilians-Universität, Munich, Germany

**Summary** Very rapid progression of disease with a median survival of 6–9 months is a common feature of metastatic cutaneous malignant melanoma. Nevertheless, substantial variability of survival suggests that metastatic cutaneous malignant melanoma can be divided into several biological subgroups. Pretreatment serum levels of soluble adhesion molecules and various clinical parameters in cutaneous metastatic malignant melanoma were evaluated to determine their prognostic value. In this study pretreatment serum levels of soluble vascular cell adhesion molecule 1 (sVCAM-1), soluble intercellular cell adhesion molecule 1 (sICAM-1), soluble endothelial leukocyte adhesion molecule 1 (sE-selectin) and multiple clinical factors were assessed in relation to overall survival of 97 consecutive patients with metastatic cutaneous malignant melanoma seen at our institution between May 1990 and April 1996. For statistical analysis, both univariate and multivariate Cox proportional-hazards models were used. Elevated pretreatment serum levels of sVCAM-1 ($P < 0.005$) and of lactate dehydrogenase ($P < 0.002$) were rendered statistically independent and were significantly associated with unfavourable outcome. Patients were assigned to one of three risk categories (low, intermediate and high) according to a cumulative risk score defined as the function of the sum of these two variables. There were significant differences in overall survival ($P < 0.0001$) between low- ($n = 53$, 5-year survival probability of 23.3%), intermediate- ($n = 29$, 5-year survival probability of 9.9%) and high-risk ($n = 15$) patients. Elevated pretreatment serum levels of sVCAM-1 and of lactate dehydrogenase correlate with poor outcome in metastatic cutaneous malignant melanoma. These data support risk stratification for future therapeutic trials and identify factors that need to be validated in prospective studies and may potentially influence decision-making in palliative management of patients with disseminated cutaneous malignant melanoma.

**Keywords:** predictor; cutaneous malignant melanoma; soluble vascular cell adhesion molecule

Over the past decade, malignant melanoma has been one of the most rapidly increasing malignancies in human (Glass and Hoover, 1989). In the United States, the number of patients with malignant melanoma nearly doubled between 1980 and 1990 (Grin-Jorgensen et al, 1992). Melanoma frequently affects young adults and is refractory to current methods of therapy once disseminated, with a median survival of 6–9 months (Legha, 1989; Koh, 1991). Nevertheless, substantial variability of survival suggests that metastatic malignant melanoma can be divided into several biological subgroups. Current methods to identify the aggressive potential of metastatic malignant melanoma are limited.

The ability of tumour cells to adhere to and to detach from extracellular matrix and endothelial cells may be crucial in tumour invasion and metastasis and may dramatically alter the clinical outcome for patients with cancer (Albelda, 1993; Gearing and Newman, 1993; Frenette and Wagner, 1996). Several univariate studies have demonstrated that elevated serum levels of soluble adhesion molecules correlate with disease progression in malignant melanoma and might serve as prognostic markers (Harning et al, 1991; Altomonte et al, 1992; Banks et al, 1993).

*Received 2 September 1997*
*Revised 3 December 1997*
*Accepted 8 December 1997*

*Correspondence to:* J Atzpodien, Department of Hematology and Oncology, Medizinische Hochschule Hannover, D-30623 Hanover, Germany

Vascular adhesion molecule 1 (VCAM-1, CD106) is a member of the immunoglobulin superfamily, expressed primarily on vascular endothelial cells and serves as a counter-receptor for the integrin $\alpha4\beta1$ (VLA-4). A soluble isoform of VCAM-1 maintains the ability to bind VLA-4 and may serve as a mediator in the metastatic process of melanoma cells (Rice and Belivacqua, 1989; Mould et al, 1994; Garofalo et al, 1995). Intercellular adhesion molecule 1 (ICAM-1, CD54) is a cell-surface molecule expressed on a variety of cell types; a soluble form of ICAM-1 (sICAM-1) is elevated in inflammation, infection and in cancers, including malignant melanoma. E-selectin, previously known as endothelial leukocyte adhesion molecule 1 (CD62e), is particularly interesting, because it is found only on activated endothelial cells, in contrast to other adhesion molecules, which have a wider tissue distribution. Endothelial cells have been shown to release E-selectin (sE-selectin) following in vitro activation (Piggot et al, 1992; Newman et al, 1993). Serum levels of sE-selectin are significantly higher in melanoma patients than in normal donors (Fortis et al, 1995).

We evaluated the prognostic value of elevated serum levels of soluble adhesion molecules sVCAM-1, sICAM-1, sE-selectin and of multiple clinical parameters in relation to overall survival in 97 consecutive patients with metastatic malignant melanoma using univariate and multivariate Cox proportional-hazards models. The purpose of this study was to identify prognostic factors for survival that may allow appropriate stratification in the management of metastatic malignant melanoma both in and outside clinical trials.

### METHODS

### Patients and collection of samples

This study was approved by the institutional review board of the Medizinische Hochschule Hannover: written informed consent was obtained from all patients before entry into the study. At that time, we obtained samples of peripheral blood from 97 consecutive patients with cutaneous metastatic malignant melanoma, seen at our institution at the Medizinische Hochschule Hannover, Germany, since May 1990. Sera were frozen at –70°C until analysis. Patient characteristics are summarized in Table 1: all patients had a Karnofsky performance status ≥70%, and presented with histologically confirmed metastatic malignant melanoma and clinically progressive disease as demonstrated by standard radiographic procedures. Patients received chemoimmunotherapy containing subcutaneous interleukin 2, interferon alpha2a, intravenous platinum and dacarbazine with or without carmustine: treatment was continued until further disease progression occurred (Atzpodien et al. 1995). Response to therapy was evaluated on an intention to treat basis and was assessed according to WHO criteria.

### Enzyme-linked immunosorbent assay (ELISA) for soluble adhesion molecules

Pretreatment levels of soluble adhesion molecules were determined using the following ELISA kits: sVCAM-1 (Medgenix, Ratingen, Germany), sICAM-1 and sE-selectin (both Quantikine, R & D, Wiesbaden, Germany). All analyses were performed in duplicate strictly according to the procedures recommended by the manufacturers and samples were analysed at a dilution resulting in measured concentrations within the range of the standard curves. Normal donor sera were obtained from healthy control subjects ($n$ = 10); mean serum values ± s.e.m. for sVCAM-1, sICAM-1 and sE-selectin concentrations of the healthy control group were 509 ng $ml^{-1}$ ± 14 ng $ml^{-1}$, 208 ng $ml^{-1}$ ± 12 ng $ml^{-1}$, and 54 ng $ml^{-1}$ ± 7 ng $ml^{-1}$ respectively.

### Statistical analysis

The statistical end point in our analysis was overall survival from time of entry into the study. We calculated univariate hazard ratios with 95% confidence intervals, using the Cox proportional-hazards model (Cox, 1972). The simultaneous prognostic effect of various factors was determined in a multivariate analysis using the Cox proportional-hazards model (forward selection of variables). The probability of overall survival was plotted over time according to the method of Kaplan and Meier (Kaplan and Meier, 1958). Differences between groups in overall survival were tested with Breslow statistics. Clinical parameters and soluble adhesion molecules were tested as dichotomized prognostic variables. For age, time since tumour progression, time since tumour diagnosis, erythrocyte sedimentation rate (ESR), neutrophil count, haemoglobin, sICAM-1, sVCAM-1 and sE-selectin, Kaplan–Meier estimates were performed, defining the best cut-off value as the value that best discriminates between poor and good overall survival. For lactate dehydrogenase (LDH) and C-reactive protein (CRP), the institutional upper normal limits were chosen as cut-off (≤ 240 U $l^{-1}$ and < 8 mg $l^{-1}$ respectively).

**Table 1** Patient characteristics and risk categories according to elevated serum levels of soluble VCAM-1 and LDH

| Variable | Total | Low risk | Intermediate risk | High risk |
|---|---|---|---|---|
| | No. of patients | | | |
| Sex | | | | |
| Male | 61 | 33 | 18 | 10 |
| Female | 36 | 20 | 11 | 5 |
| Age (years) | | | | |
| Mean | 52.2 | 50.5 | 53.2 | 54.3 |
| Range | 20–73 | 20–71 | 23–73 | 29–73 |
| Histological subtype | | | | |
| Superficial spreading | 24 | 10 | 10 | 4 |
| Amelanotic | 16 | 7 | 3 | 6 |
| Nodular | 21 | 12 | 8 | 1 |
| Acral lentiginous | 3 | 2 | 1 | 0 |
| Unclassified cutaneous | 33 | 22 | 7 | 4 |
| Metastases | | | | |
| Skin, subcutaneous tissues, distant lymph nodes | 65 | 33 | 20 | 12 |
| Visceral metastases | 54 | 21 | 22 | 11 |
| Lung metastases | 40 | 18 | 16 | 6 |
| Brain metastases | 4 | 0 | 3 | 1 |
| Bone metastases | 9 | 4 | 4 | 1 |
| Other | 12 | 1 | 5 | 6 |

Patients were assigned to one of three risk categories (low, intermediate or high) according to cumulative risk, defined as the function of the sum of two independent variables, i.e. elevated serum levels of sVCAM-1 (≥ 770 ng $ml^{-1}$) and LDH (≥ 240 U $l^{-1}$).

### RESULTS

### Univariate analysis of pretreatment variables and survival

We analysed the ability of various clinical factors and of levels of soluble adhesion molecules to predict clinical outcome. The mean period of follow-up for the surviving patients was 21 months (range 1–67 months). The median survival of all 97 patients entering the study was 10 months.

We calculated univariate hazard ratios with the Cox proportional-hazards model (Table 2). In this analysis, LDH, ESR, the presence of visceral metastases, liver disease and the number of metastatic sites had significant prognostic value with regard to overall survival. Elevated serum levels of LDH (≥ 240 U $l^{-1}$) were present in 39 patients and were strongly associated with an unfavourable outcome: median survival in these patients was 5 months as opposed to patients with normal serum LDH ($n$ = 58), who had a median survival of 16 months ($P < 0.0001$, Figure 1). A tendency towards higher serum levels of LDH was found in patients with liver metastases ($P = 0.001$, chi-square test). Liver metastases were an important prognostic factor when examined by single-factor analysis ($P < 0.004$) and by multivariate analysis ($P < 0.04$). The median survival for patients with liver metastases was 6 months, compared with 11 months for patients without liver metastases. In general, only visceral disease was of significant prognostic value in univariate analysis ($P = 0.004$). It is notable

© Cancer Research Campaign 1998

**Table 2** Hazard ratios associated with pretreatment clinical factors and soluble adhesion molecule levels in 97 consecutive patients with disseminated cutaneous malignant melanoma

| Variable | Categories compared[a] | Hazard ratio (95%) confidence interval) | P-value[b] |
|---|---|---|---|
| *Clinical factors* | | | |
| Sex | Female vs. male | 0.99 (0.79–1.25) | 0.93 |
| Age (years) | < 60 vs ≥ 60 | 1.01 (0.80–1.28) | 0.91 |
| Time since tumour progression (months) | < 10 vs ≥ 10 | 1.17 (0.91–1.51) | 0.21 |
| Time since tumour diagnosis (months) | < 24 vs ≥ 24 | 1.05 (0.80–1.38) | 0.73 |
| Liver metastases | Absent vs present | 0.71 (0.55–0.90) | < 0.004 |
| Visceral metastases | Absent vs present | 0.69 (0.55–0.87) | 0.004 |
| Lung metastases | Absent vs present | 0.87 (0.69–1.09) | 0.2 |
| Brain metastases | Absent vs present | 0.62 (0.37–1.03) | 0.05 |
| Bone metastases | Absent vs present | 0.86 (0.59–1.28) | 0.45 |
| Skin, subcutaneous, tissue metastases | Absent vs present | 0.92 (0.73–1.17) | 0.5 |
| Metastatic sites | One site vs more than one site | 0.72 (0.57–0.91) | 0.003 |
| ESR (mm $h^{-1}$) | < 50 vs ≥ 50 | 0.67 (0.51–0.87) | 0.001 |
| CRP (mg $l^{-1}$) | < 8 vs ≥ 8 | 0.79 (0.63–1.0) | 0.05 |
| LDH (U $l^{-1}$) | < 240 vs ≥ 240 | 0.58 (0.46–0.73) | < 0.0001 |
| Neutrophil count (cells $\mu l^{-1}$) | < 6000 vs ≥ 6000 | 0.99 (0.74–1.32) | 0.91 |
| Haemoglobin (g $dl^{-1}$) | < 10 vs ≥ 10 | 1.16 (0.78–1.71) | 0.44 |
| *Adhesion molecules* | | | |
| sVCAM-1 (ng $ml^{-1}$) | < 770 vs ≥ 770 | 0.58 (0.44–0.75) | < 0.0001 |
| sICAM-1 (ng $ml^{-1}$) | < 290 vs ≥ 290 | 0.73 (0.57–0.92) | < 0.005 |
| sE-selectin (ng $ml^{-1}$) | < 60 vs ≥ 60 | 0.77 (0.61–0.97) | 0.02 |

[a]For each variable, the prognostic significance of the first category listed was assessed by comparing that category with the reference category (the second category listed). [b]For the comparison of the hazard ratio shown with a hazard ratio of 1.0 (as postulated by the null hypothesis).

that brain, lung and bone metastases were not associated with a poorer clinical outcome. Patients with brain metastases only were allowed to receive simultaneously radiation therapy to the brain. It is likely that palliative radiation therapy to the brain resulted in a long enough survival to allow other factors to be more important in predicting the clinical course of disease. Patients with a single metastasis survived longer than patients with metastases at two or more sites ($P$ = 0.003). The median survival was 14 months for patients with one metastatic site and 7 months for those with more than one metastatic site. Time since tumour progression and since tumour diagnosis was not rendered statistically significant in predicting overall survival ($P$ = 0.21 and $P$ = 0.73 respectively). Once melanoma progressed to distant metastases, there was no correlation between the sex/age of the patient and the clinical course ($P$ = 0.93 and $P$ = 0.91 respectively); survival curves were superimposable. There were no histological criteria of the primary melanomas that predicted the patients' clinical courses once they developed distant metastases.

All adhesion molecules tested had significant prognostic value. Elevated serum levels of sVCAM-1 (≥ 770 ng $ml^{-1}$, $n$ = 20) were associated with the highest likelihood of an unfavourable outcome. The median survival for these patients was very poor (5 months) as opposed to patients in whom no elevated sVCAM-1 levels were observed (median survival 12 months, $n$ = 57, $P$ < 0.0001, Figure 2). Similarly, patients with elevated serum levels of sICAM-1 (≥ 290 ng $ml^{-1}$, $n$ = 56, median survival, 6 months) and patients with elevated serum levels of sE-selectin (≥ 60 ng $ml^{-1}$, $n$ = 32, median survival, 6 months) had a reduced overall survival when compared with patients without elevated sICAM-1 and sE-selectin respectively (median survival 16 and 13 months respectively, $P$ ≤ 0.02).

### Multivariate analysis of risk factors

To identify the most powerful prognostic factors, we established a multivariate Cox proportional-hazards model containing those factors with significant prognostic value upon univariate analysis. The following three factors were found to be significant: liver metastases ($P$ < 0.04), serum LDH ($P$ < 0.002) and sVCAM-1 ($P$ < 0.005). The hazard ratios calculated with a model containing these prognostic factors are shown in Table 3. Factors also evaluated and found not to be independent by multivariate analyses included evaluated ESR, visceral disease, more than one metastatic site, sICAM and sE-selectin. These results were the same even after accounting for palliative chemotherapy. This complex model resulted in numerous prognostic subgroups of small sizes and, therefore, it appeared inappropriate for further statistical verification.

### Development of a cumulative risk model

On the basis of those two pretreatment prognostic parameters of highest statistical significance, which were rendered independent upon multivariate analysis, i.e. elevated serum levels of sVCAM-1 and LDH, patients were assigned to one of three risk categories: low risk, defined as the absence of either risk factor; intermediate risk, defined as the presence of one risk factor; and high risk, defined as the presence of both risk factors (Figure 3). There were significant differences in overall survival ($P$ < 0.0001) between low-risk ($n$ = 53, median survival 16 months, 5-year survival probability 23.3%), intermediate-risk ($n$ = 29, median survival 6 months, 5-year survival probability 9.9%) and high-risk patients ($n$ = 15, median survival 5 months, 5-year survival probability

© Cancer Research Campaign 1998

**Figure 1** LDH and survival in 97 patients with metastatic cutaneous malignant melanoma. Survival curves (Kaplan–Meier estimate) of patients with either low (< 240 U l⁻¹) or elevated (≥ 240 U l⁻¹) serum levels of LDH. P-value was determined by log-rank test. Tick marks represent patients for whom data were censored

0%). The intermediate risk group consisted of five patients with elevated sVCAM-1 levels and 24 patients with elevated LDH. The median survival for both subgroups showed no statistically significant difference. None of the metastatic cutaneous malignant melanoma patients at high risk survived for more than one year.

### DISCUSSION

Our study found that elevated pretreatment serum sVCAM-1 is a potentially powerful prognostic indicator of overall survival in patients with metastatic cutaneous malignant melanoma. By univariate analysis, the most significant prognostic variables were (1) serum LDH, (2) erythrocyte sedimentation rate, (3) the presence of visceral metastases, (4) the presence of liver metastases, (5) more than one metastatic site and (6) elevated serum levels of soluble adhesion molecules VCAM-1, ICAM-1 and E-selectin.

Multivariate analysis showed that elevated serum levels of sVCAM-1 ($P < 0.005$) and of LDH ($P < 0.002$) were the dominant independent prognostic variables. When combining elevated sVCAM-1 and serum LDH we were able to identify a subgroup of patients at highest risk, all of whom died of disease within 1 year. In contrast, intermediate-risk patients exhibiting one of the two pretreatment risk factors reached a 5-year survival probability of 10%, whereas median survival was similar to high-risk patients. In comparison with high- and intermediate-risk patients, the subgroup at lowest risk demonstrated a median survival of 16 months and a 5-year survival probability of 23%. This risk stratification suggested that metastatic cutaneous malignant melanoma patients can be divided into several biological subgroups; however, the present model needs to be validated in prospective trials.

LDH has been reported as a prognostic parameter in metastatic malignant melanoma, in correlation with tumour burden (Heimdal et al, 1989; Sirott et al, 1993). An elevated serum LDH does not necessarily indicate liver metastasis. In this study, 40% of patients had elevated serum levels of LDH, but only 26% had confirmed liver metastases. Moreover, serum LDH and the presence of liver metastases were independent prognostic markers upon multivariate analysis. The reduced survival of patients with more than one metastatic site confirms the findings of Balch et al (1983). However, in the present study the number of metastatic sites was not of independent significance. This may be explained in part by the inclusion of serum LDH in this study, which achieved statistical significance as a more accurate marker of tumour burden. Erythrocyte sedimentation rate, which is a known unspecific marker in various human malignancies, did not reach statistical independence in the prognosis of metastatic malignant melanoma.

We were able to confirm and extend earlier reports of elevated serum levels of sICAM-1 and sE-selectin in metastatic malignant melanoma patients. Although in this multivariate analysis, sE-selectin and sICAM-1 were of no prognostic value, sVCAM-1 presented as an independent predictor of survival for patients with

**Figure 2** sVCAM-1 and survival in 97 patients with metastatic cutaneous malignant melanoma. Survival curves (Kaplan–Meier estimate) of patients with either low (< 770 ng ml⁻¹) or elevated (≥ 770 ng ml⁻¹) serum levels of sVCAM-1. P-value was determined by log-rank test. Tick marks represent patients for whom data were censored

**Figure 3** Kaplan–Meier curves of patient categories stratified according to cumulative risk of elevated pretreatment serum sVCAM-1 and LDH in 97 patients with metastatic cutaneous malignant melanoma. The study population of patients with metastatic cutaneous malignant melanoma was analysed according to elevated pretreatment serum levels of sVCAM-1 and LDH. Patients were assigned to one of three risk categories according to cumulative risk, defined as the function of the sum of two independent variables, i.e. elevated serum levels of sVCAM-1 (≥ 770 ng ml⁻¹) and LDH (≥ 240 U l⁻¹). P-value was determined by log-rank test. Tick marks represent patients for whom data were censored

Table 3 Hazard ratios associated with pretreatment prognostic factors in a multivariate analysis of 97 consecutive patients with cutaneous malignant metastatic melanoma, using Cox proportional-hazards models

| Variable[a] | Categories compared[b] | Hazard ratio (95% confidence interval) | P-value[c] |
|---|---|---|---|
| *Clinical factors* | | | |
| Liver metastases | Absent vs present | 0.76 (0.59–0.97) | < 0.04 |
| LDH (U $l^{-1}$) | < 240 vs ≥ 240 | 0.61 (0.48–0.79) | < 0.002 |
| *Adhesion molecules* | < 770 vs ≥ 770 | 0.71 (0.54–0.95) | < 0.005 |
| sVCAM-1 (ng $ml^{-1}$) | | | |

[a]Factors also evaluated and rendered not independent by multivariate analysis included elevated ESR, more than one metastatic site, visceral metastases, sICAM-1 and sE-selectin. [b]For each variable, the prognostic significance of the first category listed was assessed by comparing that category with the reference category (the second category listed). [c]For the comparison of the hazard ratio shown with a hazard ratio of 1.0 (as postulated by the null hypothesis).

metastatic cutaneous malignant melanoma. Notably, in the present study, pretreatment elevation of sVCAM-1 was most probably not due to tumour burden as suggested by its statistical independence upon multivariate analysis. The differences in prognostic value with regard to sVCAM-1, sICAM-1 and sE-selectin probably reflect differences in source, kinetics of expression and signals inducing adhesion molecule shedding. The significance of adhesion molecule shedding is not clear but may have profound implications for tumour metastasis. In malignant melanoma, $\alpha 4\beta 1$ integrin (VLA-4) mediates melanoma cell adhesion and migration through binding to VCAM-1 (Mould et al, 1994; Schadendorf et al, 1995). Conversely, down-regulation of VCAM-1 on endothelial cells has been identified as a potential mechanism of melanoma escape from cytotoxic lymphocyte surveillance (Piali et al, 1995).

Several other preclinical parameters have been reported as prognostic variables in metastatic malignant melanoma. Primary tumour- or metastasis-related parameters include cytogenetic abnormalities, DNA ploidy and S-phase fraction, the expression of metastasis associated gene products, the S100 protein and the detection of circulating melanoma cells in peripheral blood using reverse transcriptase-polymerase chain reaction (RT-PCR) for tyrosinase messenger RNA (Trent et al, 1990; Smith et al, 1991; Xerri et al, 1994; Karlson et al, 1996; Kunter et al, 1996; Buer et al, 1997). Although all of these malignant melanoma-associated features reflect isolated aspects of tumour biology, in the present study we were able to define a new and highly specific parameter potentially indicative of tumour cell–extracellular matrix interaction and its impact on survival in malignant melanoma.

In conclusion, elevated pretreatment serum levels of sVCAM-1 and of LDH correlate with poor outcome in cutaneous metastatic malignant melanoma. These data support risk stratification for future therapeutic trials, and identify factors that may potentially influence decision-making in palliative management of patients with disseminated malignant melanoma. In addition, an improved understanding of the endothelial matrix-associated mechanisms of tumour cell adhesion and invasion may provide a lead for future therapeutic strategies focusing on the regulation of endothelial function and damage in human malignancies. Our results will have to be confirmed prospectively in future controlled studies.

### REFERENCES

Albelda SM (1993) Role of integrins and other cell adhesion molecules in tumor progression and metastasis. *Lab Invest* **68**: 5–17

Altomonte M, Colizzi F, Esposito G and Maio M (1992) Circulating intercellular adhesion molecule 1 as a marker of disease progression in cutaneous melanoma. *N Engl J Med* **327**: 959

Atzpodien J, Lopez Hänninen E, Kirchner H, Franzke A, Körfer A, Volkenandt M, Duensing S, Schomburg A, Chaitchik S and Poliwoda H (1995) Chemoimmunotherapy of advanced malignant melanoma: sequential administration of subcutaneous Interleukin-2 and Interferon-a after intravenous dacarbazine, cisplatin, carmustine and Tamoxifen. *Eur J Cancer* **31A**: 876–881

Balch CM, Soong SJ, Murad TM, Smith JW, Maddox WA and Durant JR (1983) A multifactorial analysis of melanoma. IV. Prognostic factors in 200 melanoma patients with distant metastases (stage III). *J Clin Oncol* **1**: 126–134

Banks RE, Gearing AJH, Hemingway IK, Norfolk DR, Perren TJ and Selby PJ (1993) Circulating intercellular adhesion molecule-1 (ICAM-1), E-selectin and vascular cell adhesion molecule-1 (VCAM-1) in human malignancies. *Br J Cancer* **68**: 122–124

Buer J, Probst M, Franzke A, Duensing S, Haindl J, Volkenandt M, Ganser A and Atzpodien J (1997) Elevated serum levels of S100 and survival in metastatic malignant melanoma. *Br J Cancer* (in press).

Cox DR (1972) Regression models and life-tables. *J R Stat Soc (B)* **34**: 187–220

Fortis C, Galli L, Consogno G, Citterio G, Matteucci P, Scaglietti G and Bucci E (1995) Serum levels of soluble cell adhesion molecules (ICAM-1, VCAM-1, E-Selectin) and of cytokine TNF-α increase during interleukin-2 therapy. *Clin Immunol Immunopathol* **75**: 142–147

Frenette PS and Wagner DD (1996) Adhesion molecules – part I. *New Engl J Med* **334**: 1526–1529

Garofalo A, Chirivi RGS, Foglieni C, Pigott R, Mortarini R, Martin-Padura I, Anichini A, Gearing AJ, Sanchez-Madrid F, Dejana E and Giavazzi R (1995) Involvement of the very late antigen 4 integrin on melanoma in interleukin 1-augmented experimental metastasis. *Cancer Res* **55**: 414–419

Gearing ALH and Newman W (1993) Circulating adhesion molecules in disease. *Immunol Today* **14**: 506–512

Glass AG and Hoover RN (1989) The emerging epidemic of melanoma and squamous cell cancer. *JAMA* **262**: 2097–2100

Grin-Jorgensen CM, Rigel DS, Friedman RJ (1992) The worldwide incidence of malignant melanoma. In *Cutaneous Melanoma*, Balch MB, Houghton AN, Milton GW, Sober AJ, Soong SJ (eds), pp. 27–39. JB Lippincott: Philadelphia

Harning R, Mainolfi E, Bystryn J-C, Henn M, Merluzzi VJ and Rothlein R (1991) Serum level of circulating intercellular adhesion molecule 1 in human malignant melanoma. *Cancer Res* **51**: 5003–5005

Heimdal K, Hannisdal E and Gunderson S (1989) Metastatic malignant melanoma. *Eur J Cancer* **25**: 1219–1223

Kaplan EL and Meier P (1958) Nonparametric estimation from incomplete observations. *J Am Stat Assoc* **53**: 457–481

© Cancer Research Campaign 1998

Karlsson M. Jungnelius U. Aamdal S. Boeryd B. Carstensen J and Kågedal B (1996) Correlation of DNA ploidy and S-phase fraction with chemotherapeutic response and survival in a randomized study of disseminated malignant melanoma. *Int J Cancer* **65**: 1–5

Koh HK (1991) Cutaneous melanoma. *N Engl J Med* **325**: 171–182

Kunter U. Buer J. Probst M. Duensing S. Dallmann I. Grosse J. Kirchner H. Schluepen EV. Volkenandt M. Ganser A and Atzpodien J (1996) Peripheral blood tyrosinase messenger RNA detection and survival in malignant melanoma. *J Natl Cancer Inst* **88**: 590–594

Legha SS (1989) Current therapy for malignant melanoma. *Semin Oncol* **16**: 34–44

Mould AP. Askari JA. Craig SE. Garratt AN. Clements J and Humphries MJ (1994) Integrin α4β-mediated melanoma cell adhesion and migration on vascular cell adhesion molecule-1 (VCAM-1) and the alternatively spliced IIICS region of fibronectin. *J Biol Chem* **44**: 27224–27230

Newman W. Beall LD. Carson CW. Hunder GG. Graben N. Randhawa ZI. Gopal TV. Wiener-Kronisch J and Matthy MA (1993) Soluble E-selectin is found in the supernatant of activated endothelial cells and is elevated in the serum of patients with septic shock. *J Immunol* **150**: 644–654

Piali L. Fichtel A. Terpe HJ. Imhof BA and Gisler RH (1995) Endothelial vascular cell adhesion molecule 1 expression is suppressed by melanoma and carcinoma. *J Exp Med* **181**: 811–816

Pigott R. Dillon LP. Hemingway ICH and Gearing AJH (1992) Soluble forms of E-selectin. ICAM-1 and VCAM-1 are present in the supernatant of cytokine-activated endothelial cells. *Biochem Biophys Res Commun* **187**: 584–589

Rice GE. Belivacqua MP (1989) An inducible endothelial cell surface glycoprotein mediates adhesion. *Science* **246**: 1303–1306

Schadendorf D. Heidel J. Gawilk C. Suter L and Czarnetzki BM (1995) Association with clinical outcome of expression of VLA-4 in primary cutaneous malignant melanoma as well as P-selectin and E-selectin on intratumoral vessels. *J Natl Cancer Inst* **87**: 366–371

Sirott MN. Bajorin DF. Wong GYN. Tao Y. Chapman PB. Templeton MA and Houghton AN (1993) Prognostic factors in patients with metastatic malignant melanoma. *Cancer* **72**: 3091–3098

Smith H. Selby P. Southgate J. Pittman K. Bradley C and Blair GF (1991) Detection of melanoma cells in peripheral blood by means of reverse transcriptase and polymerase chain reaction. *Lancet* **338**: 1227–1279

Trent JM. Meyskens FL. Salmon SE. Ryschon K. Leong SPL. Davis JR and McGee DL (1990) Relation of cytogenetic abnormalities and clinical outcome in metastatic melanoma. *New Engl J Med* **322**: 1508–1511

Xerri L. Grob JJ. Battyani Z. Gouvernet J. Hassoun J and Bonerandi JJ (1994) NM23 expression in metastasis of malignant melanoma is predictive prognostic parameter correlated with survival. *Br J Cancer* **70**: 1224–1228

*© Cancer Research Campaign 1998*